# ‘What does good look like’—exploring access to healthcare for the homeless population in Gateshead, England

**DOI:** 10.1093/pubmed/fdad020

**Published:** 2023-04-12

**Authors:** Sadie Perkin, Shelina Visram, Laura Lindsey

**Affiliations:** Public Health Team, Gateshead Council, Civic Centre, Regent Street, Gateshead NE8 1HH, UK; Population Health Sciences Institute, Newcastle University, Claremont Road, Newcastle upon Tyne NE1 7RU, UK; School of Pharmacy, Newcastle University, King’s Road, Newcastle upon Tyne NE1 7RU, UK

**Keywords:** access to healthcare, health inequalities, homelessness, interviews, qualitative research, support workers

## Abstract

**Background:**

individuals who are homeless encounter extreme health inequalities and as a result often suffer poor health. This study aims to explore ways in which access to healthcare could be improved for individuals who are homeless in Gateshead, UK.

**Methods:**

twelve semi-structured interviews were conducted with people working with the homeless community in a non-clinical setting. Transcripts were analysed using thematic analysis.

**Results:**

six themes were identified under the broad category of ‘what does good look like’, in terms of improving access to healthcare. These were: facilitating GP registration; training to reduce stigma and to provide more holistic care; joined-up working in which existing services communicate rather than work in isolation; utilising the voluntary sector as support workers could actively support access to healthcare and provide advocacy; specialised roles such as specialised clinicians, mental health workers or link workers; and specialised bespoke services for the homeless community.

**Conclusions:**

the study revealed issues locally for the homeless community accessing healthcare. Many of the proposed actions to facilitate access to healthcare involved building upon good practice and enhancing existing services. The feasibility and cost-effectiveness of interventions suggested requires further assessment.

## Introduction

Homelessness is typically viewed as an issue relating to housing and social care, but there is increasing evidence it is also a public health issue.^1^ People who are homeless are considered to be one of the populations experiencing the poorest health in society, frequently suffering from a trimorbidity of physical health, mental health and substance misuse issues.^2^ The 2022 Homeless Health Needs Audit revealed that 77% of respondents reported a physical health condition, 82% a mental health diagnosis and 38 and 29% reporting drug and alcohol addiction, respectively.^1^ The average age of death amongst people who are homeless in England and Wales between 2013 and 2019 was 43 years in women and 46 years in men, significantly lower than the general population (81 and 76 years, respectively).^3^

The NHS constitution states that NHS care is to be provided to all based on clinical need irrespective of an individual’s background.^4^ The homeless population face extreme health inequalities in both health outcomes and access to healthcare.^5^ In accordance to the inverse care law, people who are homeless are 40 times more likely to be unregistered with a GP than a housed member of the population.^2^ Use of A&E is often high amongst individuals who are homeless and a study in Birmingham revealed their rate of A&E attendances to be almost 60 times that of the general population.^6^

The study described in this paper was conducted in Gateshead, a metropolitan borough in the North East of England with a population of ~202 500. It is the 47th most deprived of the 317 local authority areas in England. Gateshead has the second highest rate of homelessness in the North East, with 145 people estimated to be rough sleeping or living in temporary accommodation in 2019.^7^

Local audits within Gateshead highlighted issues accessing healthcare amongst people who are homeless, with high proportions of individuals wanting to access GP, mental health and addiction services reportingly finding it impossible or difficult to do so (70, 89 and 67%, respectively).^8^ Also, 66% of individuals presenting to the local drop-in centre self-reported not being registered with a GP, whereas 79% reported having accessed A&E in the past year.^9^ At the time of conducting this study, there was no specialist general healthcare provision for individuals who are homeless within Gateshead. This qualitative study aimed to explore access to healthcare for individuals who are homeless in Gateshead, with an emphasis on what good provision would look like.

## Method

### Methodology

**Fig. 1:**
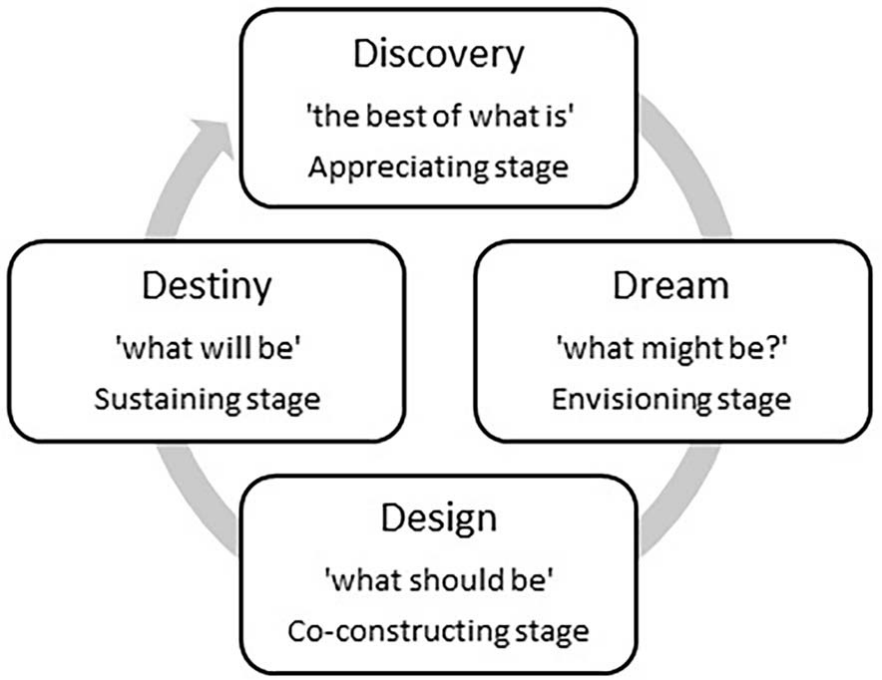
Stages of AI: the 4D model.^10^

This study was influenced by an appreciative inquiry (AI) approach, which is a methodology incorporating action research and organisational change.^10^ AI focuses on strengths and positive experiences to facilitate motivation, change and development, and is therefore well suited to topics where challenges and negative experiences are well established.^11^ The four stages of AI are presented in Fig. 1. Only the first two, discovery and dream, were completed in this study because of time constraints and restrictions imposed by the COVID-19 pandemic.

### Sampling and recruitment

Purposive sampling was used to identify participants with insights relevant to the research aim.^12^ Inclusion criteria were: having experience working directly or indirectly with the local homeless community, being able to communicate in English and being aged 18 or over. Participants were identified through the lead researcher’s professional networks as a public health registrar in Gateshead.

A sample size of 10–15 participants was considered to be feasible and sufficient in terms of reaching data saturation. Twenty-two individuals were invited by e-mail to participate in the study. Fifteen responded and 12 interviews were conducted (following difficulties in contacting or arranging interviews with the other three).

### Data collection

Data were collected using semi-structured interviews to allow the necessary topics to be discussed, but with scope for variation between interviews.^13^ A topic guide was developed based on existing literature and focused on generalised access for primary care, A&E, mental health and drug and alcohol services. It included probes on existing barriers and facilitators, as well as envisioning feasible changes to improve access. The questions utilised an appreciative mindset, which attempts to understand what we need more of rather than what we want less of.^11^

Data collection occurred during July and August of 2020 and because of the COVID-19 restrictions during this time interviews were conducted over Microsoft Teams or Zoom. Zoom provided an automated transcription, which was used as the starting point for transcription, whereas Microsoft Teams interviews required full transcription.

### Analysis

Data were analysed using thematic analysis employing the one-sheet-of-paper (OSOP) method, which involves mapping emerging codes, themes and categories on a single piece of paper.^14^ Coding was performed by the lead researcher in discussion with co-authors and began during the transcription process. Following the use of OSOP, codes and potential themes were written on Post-It Notes and arranged visually. Emerging themes were then entered into Microsoft Excel to further review, rearrange, name and define themes and sub-themes, which were discussed with other team members. As a form of respondent validation, the themes were circulated to all participants to ensure the researchers’ interpretations reflected the local situation. No participants raised issues with the analysis.

### Ethics

Ethical approval was granted by Newcastle University Faculty of Medical Sciences Research Ethics Committee (Ref. 4118/2020). Participants received an information sheet and provided written consent via an online form.

## Results

Eight participants worked in the voluntary sector (five support workers, one manager and two in combined roles), three worked for the local authority and one worked in primary care management. No demographic data were collected but eight of the 12 presented as female.

This paper focuses on the identified themes relating to the category of ‘what does good look like’. These were: facilitate primary care registration, training, joined-up working, utilising the voluntary sector, specialised roles and bespoke offer. The analysis also identified a number of themes that were categorised as barriers and facilitators to accessing healthcare, which are not reported here.

### Facilitate primary care registration

Access to general practice for people experiencing homelessness was considered vital, in terms of encouraging appropriate use of both primary care and acute services.

‘They’ll turn up for Accident & Emergency when it’s not an accident or an emergency […] with things that they should be going to the GP with. And I do think that potentially in cracking one nut you then crack another […] Sort out access to primary care services, essentially you ease quite a lot of the burden on A&E.’ (Participant 9)

Participants described difficulties encountered by individuals who are homeless while attempting to register with a GP; these often related to issues around lack of proof of address or identification.

‘they tried to come and register and were told “no you can’t be registered because you’re homeless” […] that’s about as bad as it gets, you know, if you’ve just been turned away and you can’t access health care.’ (Participant 3)

Options to facilitate registration include using the third sector drop-in centre as a ‘care of’ address and educating practices to allow registration of individuals despite a lack of identification or proof of address, as per NHS guidelines.

### Training

A greater understanding of homelessness and knowledge of multiple complex needs amongst healthcare workers were perceived to facilitate access to healthcare and improve attitudes towards these patients.

‘I know a couple of practices who really encourage their receptionists and their doctors to be kind of trauma-informed, so in order to understand the effects of alcohol use, or how their mental health might present […] A lot of people that we do have on the streets have suffered some really horrific situations in their life […] knowing then that leads to sometimes a lapse in their behaviour and their judgement.’ (Participant 11)

Participants felt the care provided to individuals who are homeless should take a holistic approach and be trauma-informed, ‘rather than just knowing the medical bit’ (Participant 11).

‘There’s something there about how people address trauma in a safe way, but also in an accessible way that maybe doesn’t run away from drugs always or doesn’t always run away from chaos.’ (Participant 4)

There was felt to be a particular lack of understanding of dual diagnosis and lack of recognition that drugs and alcohol are frequently used as a form of self-medication for mental health issues and/or trauma. Improving this understanding could facilitate access to mental health services for individuals with co-existing substance abuse issues.

‘[The] vast majority of substance misuse problems, they are either self-medicating for actually being homeless… but also the contributing factors which led them to get there in the first place. So that would be adverse childhood experiences, poverty, deprivation, unmet need.’ (Participant 6)

### Joined-up working

Related to the previous theme, participants perceived joined-up working to be important in improving access to healthcare because of overlap in the needs of service users.

‘holistic provision for the person that sees the needs as interconnected rather than fragmented. So, the social care needs aren’t separate from the heath needs which aren’t separate from the support with crime prevent which aren’t separate from the needs with addiction.’ (Participant 10)

There was recognition of good work within different areas of healthcare but it was felt that greater communication between teams and services would improve care. Participants described how individuals often have to repeat themselves and are bounced between services.

‘everybody is brilliant in their own pocket, but sometimes it feels like nobody’s working together.’ (Participant 5)

‘they [services] are working with the same person in the middle, and this person just […] keeps going round and round and round in a circle bouncing off each service, never kind of getting any further forward and it just seems like it’s staring you in the face […] communicate with each other.’ (Participant 7)

### Embrace the voluntary sector

Many of the participants were support workers and described strong and trusting relationships with their service users. The support workers were dedicated to being a safe haven for individuals who are homeless and persevered to maintain relationships regardless of how difficult this could be at times.

‘I think she trusts us, we are a familiar face you know, and I think she knows we are there to help her and that we aren’t judging her […] we have persevered and […] there have been times when she has kind of been really awful to us and sort of shouting and swearing and we’ve gone back the next day.’ (Participant 1)

Participants believed there was the potential for the support worker role to help facilitate healthcare access. They felt their role allowed the time and flexibility to improve access and that they could advocate for their service users and persist with disengaged individuals.

‘All statutory services […] should use the skills and resources of the voluntary sector to be able to facilitate some of the appointments and issues because we often have a lot more flexibility.’ (Participant 12)

‘people do sometimes need advocacy to get into services […] and again just the encouragement […] I think we can be helpful in that way in getting people support and giving people access.’ (Participant 10)

Recognising support workers as integral parts of multidisciplinary teams could help to improve support and advocacy for people experiencing homelessness. However, support workers felt their concerns were often dismissed because they are not health professionals and that their close relationship and understanding of the individual who is homeless is often overlooked.

‘sometimes they would be better to just listen to those of us that are working with the individuals […] it’s really difficult because we get a lot of like “you’re not health professionals” […] but we know a lot more than they think we do […] have worked in this sector for a long and time […] so we have a lot more knowledge than our lack of qualification might suggest but our opinion just isn’t really taken on board.’ (Participant 12)

### Specialised roles

There were a range of healthcare worker roles suggested by participants which might improve access to healthcare; for example, a nurse or GP who is ‘more aware of […] mental health and drug and alcohol use and any of chaotic behaviour’ (Participant 5). Several participants identified the need for a specialised mental health worker such as a community psychiatric nurse, psychotherapist or counsellor, as access to mental health services was identified as a particular concern. They also highlighted the benefit of a link worker or navigator type role, which could help to promote joined-up working by linking in with other services.

‘centralised person that can… help that individual get access to what’s out there… you’ve got that person there that can join the dots up from service to service. They’ve got that trusted relationship with the individuals but they’ve also got that trusted relationship with the services as well.’ (Participant 6)

### Bespoke offer

There was a general perception that mainstream healthcare is ‘shaped for people that are not homeless people […] people that can walk and run about their appointments’ (Participant 6)*.* A different approach was felt to be needed to improve access for the homeless population.

‘Access to health services in particular needs to be looked through a more bespoke, person-centred homelessness-shaped lens, rather than a standardised, well put-together average population-shaped lens.’ (Participant 6)

There was also a desire for a holistic, bespoke approach in which services run alongside one another to allow the multiple and complex needs of individuals experiencing homelessness to be addressed in one place.

‘All of those things [drug and alcohol, mental health, physical health] can happen in one space and potentially in one morning and right now that experience would look very different because you’re talking about months to have even one of those three encounters.’ (Participant 9)

Participants described how their service users’ behaviour could be challenging in traditional healthcare settings, likely because of previous negative experiences. Their behaviour was perceived to improve in settings in which they were comfortable. Locating healthcare in a familiar setting could therefore improve access and engagement and allow interventions to be more opportunistic.

‘At [the drop-in centre] their behaviour is completely different… whole demeanour and their whole sort of presentation and personality changes… I think it’s because it’s so relaxed… they don’t feel any threat, they feel comfortable.’ (Participant 1)

While there was a request for specialised provision, it was recognised the goal was to reintegrate individuals who are homeless back into mainstream services in the long term.

‘I think this is a means to an end. […] Eventually my hope is that they will be able to engage in a primary care service, the same way the majority of the population do. That they’ll be able to keep to a 20 past 3 appointment on a Thursday.’ (Participant 9)

## Discussion

### Main finding of this study

This study identified how access to healthcare could be improved for individuals who are homeless. Even though the study setting was a single metropolitan area in the North East of England, the applicability of the findings is not limited to this location. The main findings were that access to healthcare could be improved by: facilitating primary care registration, training, joined-up working, embracing the voluntary sector, specialised roles and a bespoke offer. The findings suggest that improving accessibility of services for people who are homeless can be achieved by a combination of breaking down barriers and drawing upon facilitators. Access to healthcare is a complex issue that requires a multifaceted approach.^15^

### What is already known on this topic

The focus of existing literature is on barriers to accessing healthcare at an individual level, such as self-esteem,^16^ complex needs^17^ and skills^18^; at provider level, such as stigma^19^ and lack of understanding^20^; and at healthcare system levels, such as registration issues,^21^ appointment systems^16^ and duration.^18^ Acknowledged facilitators to access include drop-in appointments,^17^ a multidisciplinary team approach,^19^ specialist primary care services^17^ and outreach programmes.^22^ There is increasing support for a specialised healthcare service for individuals who are homeless^2^^,^^23^; a study conducted in the UK identified 84% of individuals who were homeless preferred seeking specialised services over mainstream services.^24^ However, previous studies have not specifically addressed the potential contribution of the voluntary sector in relation to this specialist provision.

### What this study adds

There remains a paucity of literature on this subject^5^^,^^21^ and two specific gaps in the evidence shaped this study. First, no studies have been conducted on this topic exclusively in North East of England, as the focus is on areas with larger homeless populations. Consequently, the recommendations may not be appropriate, feasible or cost-effective in areas with smaller populations. As this study focuses on a local authority with a smaller homeless population; the findings may be transferable to areas with similar populations and no existing specialist homeless healthcare provision. Second, there is limited research involving staff outside the health service (e.g. in local government or the voluntary sector), despite the major role they often have in supporting people experiencing homelessness.

Many of the findings of this study related to ways to enhance current services that would not require any major restructuring. Therefore, it is likely the resources necessary to implement these findings would be minimal. The first stage involved facilitating GP registration as there were still instances of individuals being denied registration because of lack of identification and/or proof of address. Improving GP registration is crucial in enabling individuals who are homeless to access primary care and training could help disseminate the message that registration cannot be refused on the grounds of lack of identification.^25^ There are existing resources nationally that aim to combat issues around registration, for example, ‘My Right to Healthcare’ cards produced by Groundswell.^26^

Training and education could also improve knowledge and understanding of caring for patients who are homeless, particularly in terms of trauma-informed care that features in the NHS Long Term Plan.^27^ Education could also focus on holistic care as mental health, substance misuse and homelessness are not separate issues.^21^Training would potentially decrease stigma towards individuals who are homeless and ideally a degree of flexibility would be offered for these patients. Organisations such as FairHealth^28^ and Pathway^29^ provide training, which could be utilised by healthcare providers. Training could incorporate national recommendations such as that from the Care Quality Commission who suggest double appointments and a named GP for individuals who are homeless.^25^

A key finding of this study was the shared belief that if existing services worked together more efficiently then individuals who are homeless would receive better care as often individuals are bounced between services. This finding supports previous studies, which emphasised the positive role of multi-agency working.^16^^,^^19^^,^^22^ Issues such as time constraints and work pressures of staff were acknowledged as barriers to joined-up working. It was felt that the current lack of joined-up working was a result of a broken healthcare system rather than the individual staff members. Attempting to change pathways within a system can be challenging as ways of working are often embedded in organisations.^30^

Finally, a key strength of this study is the involvement of participants from the voluntary sector. These participants offered a unique stance as they wanted to advocate for their service users but were also able to reflect upon the challenges they can present to healthcare settings. Support workers often have a strong and trusting relationship with individuals who are homeless and could help with the practicalities and logistics of seeking healthcare but could also advocate for their service users. Support workers felt passionate about the health of their service users, however, felt their opinion and experience was often dismissed by healthcare providers. The Department of Health and Social Care highlights the potential role of the voluntary sector as strategic partners in addressing heath inequalities.^31^ However, it is important to note not all individuals who are homeless have contact with a support worker.

The findings did also highlight the potential for specialised roles and a bespoke offer of healthcare. However, this would likely require significant organisation and investment. This bespoke healthcare system was most often described as an opportunistic, drop-in arrangement at a location already visited by individuals who are homeless. There was also a desire for specialised roles working specifically with the homeless community including suggestions for a specialist GP or nurse, a mental health role and a link worker/navigator.

### Limitations of this study

The findings need to be interpreted mindful that they are perceptions of staff supporting the homeless community rather than those with first-hand experience of homelessness. Further work is needed to ensure that the finding of this study is reflective of individuals experiencing homelessness. Furthermore, there will be individuals, potentially the most entrenched rough sleepers or the hidden homeless, who are not accessing any services or support; it is likely their healthcare needs are even greater and the barriers even more challenging than those captured in this study. A further limitation is that the latter two stages of the AI were not completed and therefore for recommendations of this study to be implemented an assessment of feasibility and cost-effectiveness would be required.

## Conclusion

Access to healthcare for people who are homeless is a complicated issue and there is no one solution. This study proposes actions that could improve access and many of the recommendations could be implemented without delay as they would have minimal cost and would not require any major restructuring of existing services. Further work is needed in collaboration with local stakeholders, including local authority, healthcare, voluntary sector and members of the homeless population, to complete the AI and understand the feasibility and cost-effectiveness of some of the more complex interventions suggested in this study.

The NHS constitution reports a social duty to promote equality and ‘to pay particular attention to groups or sections of society where improvements in health and life expectancy are not keeping pace with the rest of the population’ (4, p3). Individuals who are homeless face extreme health inequalities^5^ and there is both a moral responsibility and legal duty; as set out by the 2012 Health and Social Care Act,^2^ to address this. This study comes at a time where the coronavirus pandemic has increased the momentum and desire for action in addressing homelessness nationally. Tackling health inequalities associated with homelessness is a colossal task but working together to improve healthcare access is a starting point on that journey.

## Conflicts of interest

At the time of conducing the research, the lead author (SP) was working in Gateshead Council as a public health registrar. Three study participants were employed by the same organisation but were not previously known to SP. The co-authors (SV and LL) have no conflicts of interest to declare.

## Funding

None.

## Data availability

Data cannot be shared for ethical/privacy reasons.

## Code availability

Not applicable.

## Authors’ contributions

All authors contributed to the study conception and design. Material preparation, data collection and analysis were performed by SP. The first draft of the manuscript was written by SP and all authors commented on subsequent versions of the manuscript. All authors read and approved the final manuscript.

## Ethics approval

Approval for the study was granted by the Faculty of Medical Sciences Research Ethics Committee at Newcastle University (Ref. 4118/2020).

## Consent to participate

All participants gave their written informed consent via an online form to participate in the study.

## Consent for publication

All participants gave written informed consent via an online form which included consent for their anonymised data to be used in publications arising from this study.

## Notes

**Sadie Perkin**, Public Health Specialty Registrar

**Shelina Visram**, Senior Lecturer

**Laura Lindsey**, Lecturer
